# Systematic expression analysis of m^6^A RNA methyltransferases in clear cell renal cell carcinoma

**DOI:** 10.1002/bco2.89

**Published:** 2021-05-03

**Authors:** Larissa Gundert, Alexander Strick, Felix von Hagen, Doris Schmidt, Niklas Klümper, Yuri Tolkach, Marieta Toma, Glen Kristiansen, Manuel Ritter, Jörg Ellinger

**Affiliations:** ^1^ Department of Urology University Hospital Bonn Bonn Germany; ^2^ Department of Pathology University Hospital Bonn Bonn Germany; ^3^ Department of Pathology University Hospital Cologne Cologne Germany

**Keywords:** ccRCC, KIAA1429, m^6^A, METTL3, METTL14, METTL4, methyltransferases, WTAP

## Abstract

**Objectives:**

To investigate the regulation of the N‐6‐methyladenosine (m^6^A) methyltransferases METTL3, METTL14, WTAP, KIAA1429, and METTL4, referred to as “m^6^A writers,” in clear cell renal cell carcinoma (ccRCC), and other RCC subtypes in respect of the potential prognostic value.

**Patients and methods:**

Tissue samples were collected within the framework of the Biobank at the Center for Integrated Oncology Bonn. The expression of the methyltransferases was systematically determined in clear cell renal carcinoma (ccRCC) on the RNA (real‐time PCR) and protein level (immunohistochemistry). Additionally, protein expression of the m^6^A writers was further investigated in papillary RCC, chromophobe RCC, sarcomatoid RCC, oncocytoma, and normal renal tissue (immunohistochemistry).

**Results:**

The expression of all m^6^A‐methyltransferases was significantly downregulated in ccRCC compared to benign renal tissue. Low m^6^A‐methyltransferase levels were correlated with higher histological grade, advanced pT‐stage, pN‐stage, and metastatic disease. Reduced m^6^A‐methyltransferase expression was associated with shorter overall survival.

**Conclusion:**

In conclusion, m^6^A‐methyltransferases are dysregulated in ccRCC and might act as tumor suppressor genes, which could be of particular importance for future diagnostic and therapeutic options.

## 1 INTRODUCTION

Kidney cancer is one of the most common malignancies in both men and women: The American Cancer Society estimated 73 750 new cases and 14 830 deaths of renal cancer in the United States for the year 2020.^1^ Renal cell carcinoma (RCC) is by far the most common form of kidney cancer, in which the clear cell renal cell subtype (ccRCC) accounts for approximately 75%–80% of RCCs.^2^ Further common subtypes are papillary renal cell carcinoma (pRCC), which represents up to 20% of RCCs,^3^ chromophobe renal cell carcinoma (chRCC), sarcomatoid renal cell carcinoma (sRCC), and renal oncocytoma. Only a few patients present with the typical symptomatic triad of a renal malignant tumor (painless hematuria, a palpable abdominal mass, and flank pain). Since early stages of RCC frequently are asymptomatic, the majority of patients is diagnosed coincidentally with suspicious renal neoplasia through abdominal or thoracic imaging of an unrelated issue^3^, ^4^; however, up to 25% of all patients with RCC are diagnosed with metastatic disease.^5^, ^6^ Gold‐standard treatment with curative intent for patients with localized RCC is partial nephron‐sparing surgery or radical nephrectomy, while treatment of patients with metastatic disease or relapse is far more complicated due to low response rates to treatment.^7^ Although there have been remarkable improvements in the overall survival of patients with metastatic RCC with the introduction of immunotherapy and targeted therapy in the form of various tyrosine kinase inhibitors, and VEGF receptor inhibitors, complete remission is rare and patients are treated with palliative intention. So far, as diagnostic and therapeutic options are limited and a metastatic stage indicates an unfavorable prognosis a more profound understanding of molecular pathologies is required to enable new genetic diagnostic procedures and personalized therapeutic options. Further development of treatments that intervene in specific targets in relevant biological pathways, such as RNA methylation and demethylation, might be yet another revolutionary step in the diagnostic and treatment of RCC.

Diverse post‐transcriptional RNA modifications have been observed. N‐6‐methyladenosine (m^6^A), discovered in the 1970s,^8^, ^9^ is the most widespread and abundant internal modification of messenger RNA (mRNA) in eukaryotic RNA.^10^ It is involved in numerous biological pathways, such as gene expression, cell growth, cell cycle, cellular differentiation and pluripotency, stem cell self‐renewal, DNA damage response, and circadian rhythm—most of which play crucial roles in cancer progression and metastasis.^9^, ^11^, ^12^, ^13^, ^14^, ^15^ The m^6^A modification is dynamically added by methyltransferases (so‐called m^6^A writers) or removed by demethylases (erasers), and mediated by RNA binding enzymes (readers). Dysregulation of these m^6^A methylases, demethylases, and RNA binding enzymes contributes to a variety of human diseases, such as obesity, diabetes, infertility, growth retardation, neurological disorders, and cancer.^14^, ^16^, ^17^, ^18^

The role of m^6^A methylases in RCC is largely unknown, and our study was designed to investigate systematically the expression of human m^6^A methyltransferases (METTL3, methyltransferase‐like protein 3; METTL4, methyltransferase‐like protein 4; METTL14, methyltransferase‐like protein 14; WTAP, Wilms‐tumor‐1 associated protein; KIAA1429, vir like m^6^A methyltransferase associated).

## 2 MATERIALS AND METHODS

### 2.1 Patients

The collection of fresh‐frozen renal tissue samples was performed within the framework of the Biobank at the Center for Integrated Oncology (CIO) Bonn as described earlier.^19^ All benign and malign tissue samples were obtained after radical or partial nephrectomy at the Department of Urology at the University Hospital Bonn. The benign samples were obtained from the adjacent non‐cancerous part of the kidneys. All patients provided written informed consent for the collection of biomaterials; the study was approved by the ethics committee of the University Bonn (approval number 127/17). The renal tissue was stored at −80°C and used for mRNA expression studies. Immunohistochemistry was conducted with archival formalin‐fixed and paraffin‐embedded tissues. All tissue samples were reviewed by a uropathologist and classified according to the WHO classification of 2009. See Supporting Information Table S1 for detailed clinicopathological parameters.

### 2.2 Quantitative real‐time PCR

Isolation of total RNA was performed with the mirVana miRNA Isolation Kit (Ambion, Foster City, CA, USA) and treated with the DNA‐free Kit (Ambion), as described earlier.^20^ The NanoDrop 2000 spectrophotometer (Thermo Scientific, Wilmington, DE, USA) was used to determine RNA quantity. RNA integrity was verified by the evaluation of the 28S and 18S ribosomal RNA bands in gel electrophoresis. We used quantitative real‐time PCR for the determination of the methyltransferases’ gene expression. cDNA was synthesized from 1 µg total RNA using the PrimeScript RT Reagent Kit with genomic DNA Eraser (Takara Bio, Saint‐Germain en‐Laye, France). PCR analyses were conducted with 2.5 ng/µL of cDNA template, SYBR Premix Ex Taq II and ROX Plus, and 10 pmol/µL of forward/reverse primer on a QuantStudio™ (Applied Biosystems by Thermo Fisher Scientific, Waltham, MA, USA). All samples were measured in triplicates. Calculation of relative gene expression levels was performed using the QuantStudio 3D Analysis Suite Cloud Software (Applied Biosystems). We used beta‐actin (ACTB), glyceraldehyde 3‐phosphate dehydrogenase (GAPDH), and peptidylprolyl isomerase A (PPIA) as reference genes, the reference gene value is an average of these three. All primer sequences are provided in Supporting Information Table S2.

### 2.3 Immunohistochemistry

The expression of the methyltransferases was further investigated in ccRCC, papillary RCC (pRCC), chromophobe RCC (chRCC), sarcomatoid RCC (sRCC), oncocytoma, and benign renal parenchyma using a tissue microarray. Three tissue cores were arrayed to obtain a representative image of each tumor; see Supporting Information Table S3 for detailed clinicopathological data. The tissue microarray with formalin‐fixed, paraffin‐embedded archival tissues was cut 5 µm thick sections, placed down in a water bath at 45°C for ideal expansion, applied on slides, and dried at 65°C for 60 minutes. Afterward, the slides were loaded in the Benchmark Ultra system (Ventana Medical Systems Inc, Illkirch, France), in which the automated processes of deparaffinization, pretreatment with cell conditioning buffer (CC1 buffer, pH 8), and incubation with the primary antibodies (METTL3 1:1000; METTL14 1:100; WTAP 1:100; KIAA1429 1:10; METTL4 1:50) at 37°C for 36 minutes proceeded. Signal detection was performed with the HRP Multimer technology of the UltraView DAB IHC Detection Kit (Ventana) and finally, the slides were counterstained using Mayer's hemalum and bluing reagent (Ventana). The staining intensity was evaluated using QuPath software.^21^ A representative immunohistochemistry is shown in Supporting Information Figure S1.

### 2.4 Statistical analyses

Statistical analyses (Kolmogorov–Smirnov test, *t* test, Kruskal–Wallis test *H*‐test, Mann–Whitney *U*‐test, Spearman‐rank correlation analyses, Bootstrap analysis, univariate and multivariate Cox regression analyses, Kaplan–Meier estimates) were performed, as appropriate, with the Statistical Package for Social Sciences (SPSS®), version 25 (SPSS INC., IBM Corp., Armonk, NY, USA). Statistical significance was concluded at *P* < .05. Cut‐off values used for survival analysis were determined using receiver operator characteristic curve analysis.

## 3 RESULTS

### 3.1 mRNA expression of m^6^A methyltransferases in ccRCC

**FIGURE 1:**
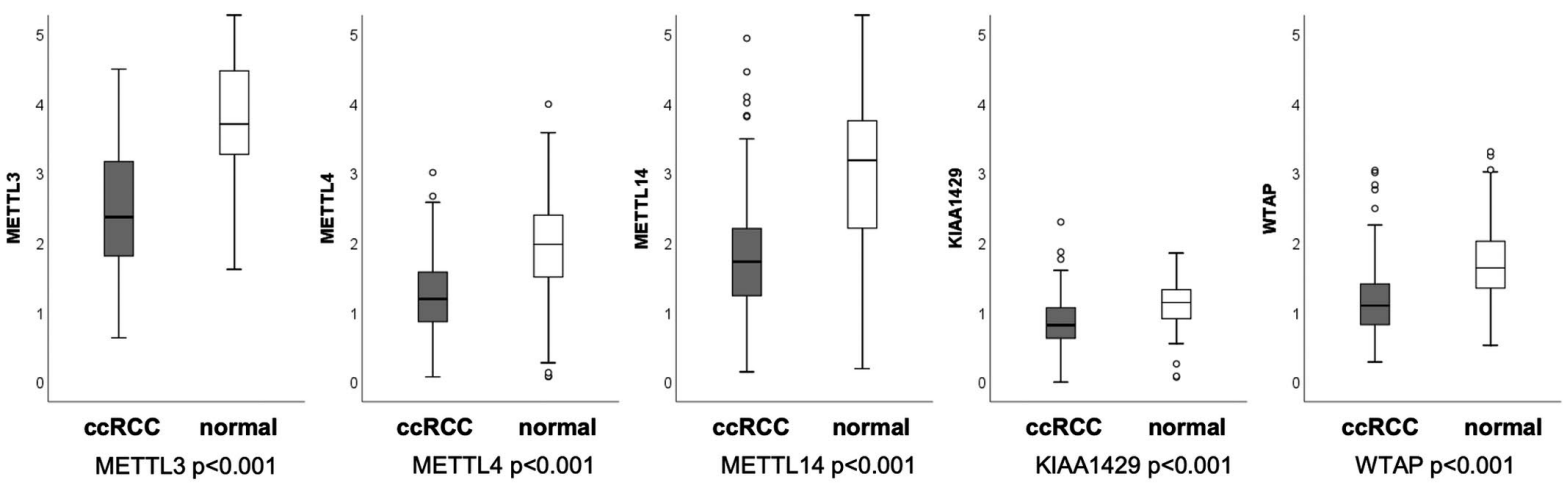
mRNA expression of m^6^A‐methyltransferases in ccRCC compared to normal renal tissue. mRNA expression of m^6^A‐methyltransferases is depleted in ccRCC compared to normal tissue

The expression of the m^6^A‐methyltransferases was investigated in ccRCC (n = 166) and normal renal tissues (n = 102). All m^6^A‐methyltransferases were significantly downregulated (Mann–Whitney *U*‐test, all *P* < .001) in ccRCC compared to adjacent renal tissue, see Figure 1. Spearman's rank correlation evinced significant correlations between all of the methyltransferases (all *P* < .001). Lower mRNA expression levels were correlated with adverse clinical‐pathological parameters (Mann–Whitney *U*‐test, Kruskal–Wallis *H*‐test): histological grade (METTL3 G1/2 vs. G3/4, *P* = .01), advanced pT‐stage (METTL4 pT1/2 vs. pT3/4, *P* = .039), and distant metastasis (METTL3 *P* = .043; METTL4 *P* = .05; METTL14 *P* = .003; KIAA1429 *P* = .024; WTAP *P* = .004).

**FIGURE 2:**
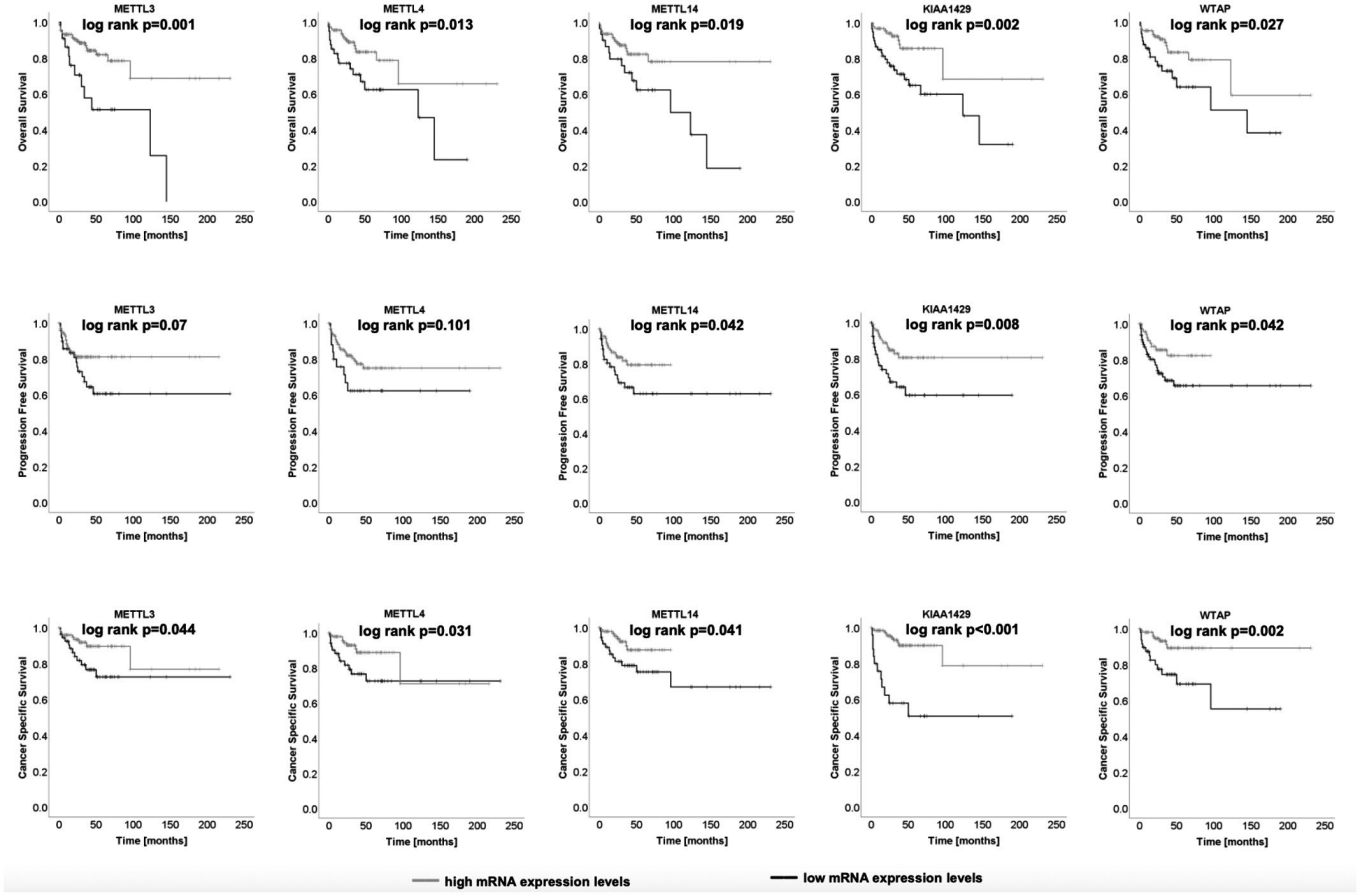
Kaplan–Meier estimates indicate a poor outcome in patients with ccRCC and low m^6^A methyltransferases mRNA levels

**FIGURE 3:**
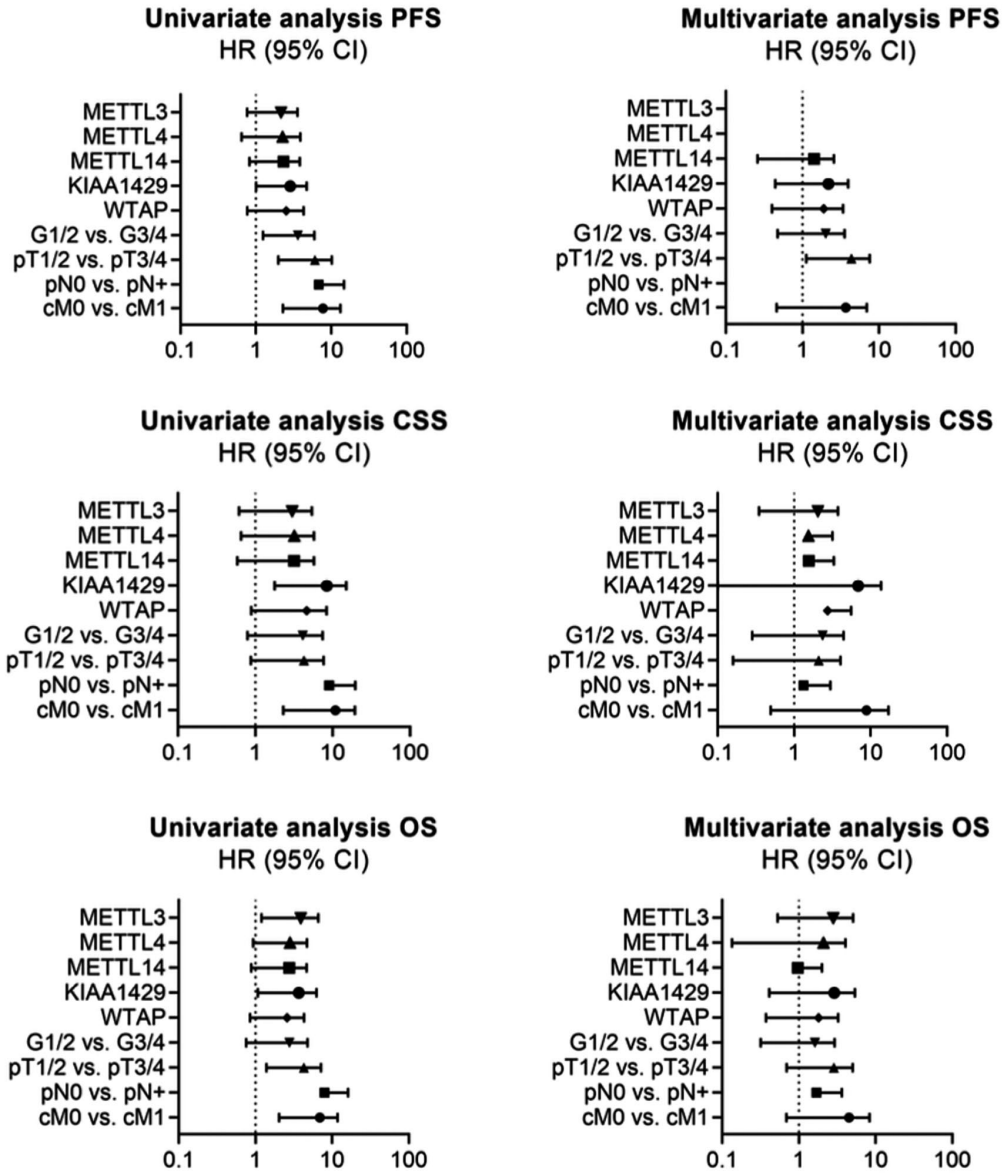
Forest plot analyses of univariate and multivariate Cox regression analyses of the PCR cohort for prediction of progression‐free survival, cancer‐specific survival, and overall survival

Kaplan–Meier estimates indicated that decreased m^6^A methyltransferase expression was predictive of poor outcome in ccRCC patients (see Figure 2): progression‐free survival (PFS; METTL14 log‐rank *P* = .0042; KIAA1429 log‐rank *P* = .008; WTAP log‐rank *P* = .042), cancer‐specific survival (CSS; METTL3 log‐rank *P* = .044; METTL4 log‐rank *P* = .031; METTL14 log‐rank *P* = .041; KIAA1429 log‐rank *P* < .001; WTAP log‐rank *P* = .002), and overall survival (OS; METTL3 log‐rank *P* = .001; METTL4 log‐rank *P* = .013; METTL14 log‐rank *P* = .019; KIAA1429 log‐rank *P* = .002; WTAP log‐rank *P* = .027) were shortened in patients with low m^6^A‐methyltransferase expression levels; see Supporting Information Tables S4‐6 for details. Also, univariate Cox regression analysis demonstrated the prognostic value of the methyltransferases in patients with ccRCC, low expression meaning poor overall survival (all *P* < .05), shortened cancer‐specific (all *P* < .05, except for METTL3 *P* = .051, which still showed a strong tendency toward reduced CSS), and shortened progression‐free survival (METTL14 *P* = .046; KIAA1429 *P* = .01; WTAP *P* = .047). However, statistical significance was not observed in the multivariate Cox regression analysis. Parameters that did not show statistical significance in the univariate model (PFS, METTL3, and METTL4, both *P* > .05) were not included in the multivariate model; see Figure 3 and Supporting Information Table S7‐9. We reviewed our findings with analysis of the bias‐corrected and accelerated (BCa) bootstrap interval for progression‐free survival, cancer‐specific survival, and overall survival; for detailed information see Supporting Information Table S10‐12.

### 3.2 Validation of m^6^A methyltransferases expression using the TCGA dataset

**FIGURE 4:**
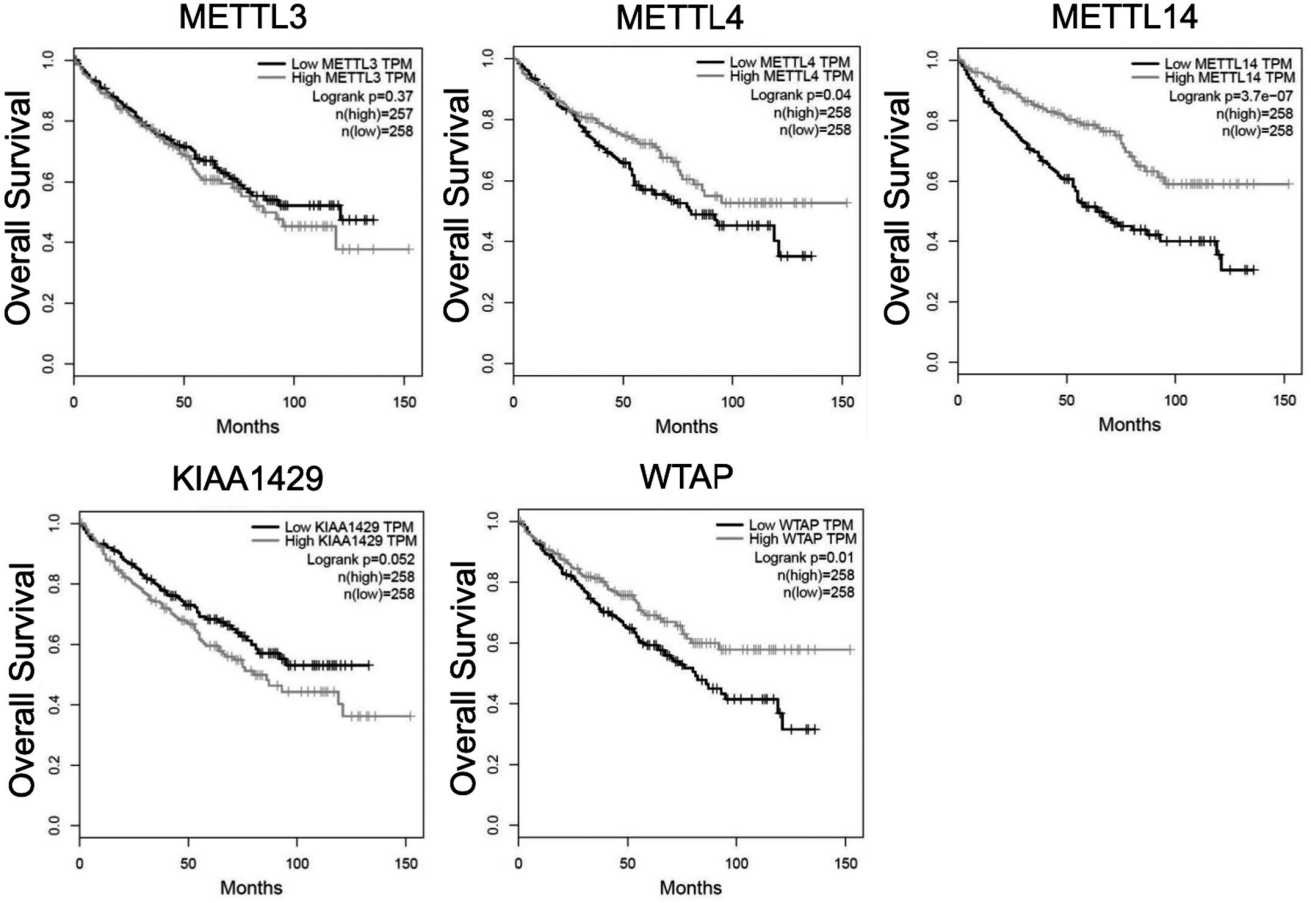
Kaplan–Meier estimates derived from the TCGA dataset confirm poor outcome in patients with ccRCC and low METTL4, METTL14, KIAA1429, and WTAP expression levels

We used the data generated by The Cancer Genome Atlas (TCGA) Research Network (https://www.cancer.gov/tcga) to validate our findings, and GEPIA (Gene Expression Profiling Interactive Analysis)^22^ was used to create Kaplan–Meier estimates. The expression of METTL4 (log‐rank *P* = .004), METTL14 (log‐rank *P* < .001), and WTAP (log‐rank *P* = .01) was associated with shorter overall survival. KIAA1429 showed a strong tendency toward a shortened overall survival (log‐rank *P* = .052), while METTL3 was not correlated with overall survival (log‐rank *P* = .37); see Figure 4.

### 3.3 Protein expression of m^6^A methyltransferases

**FIGURE 5:**
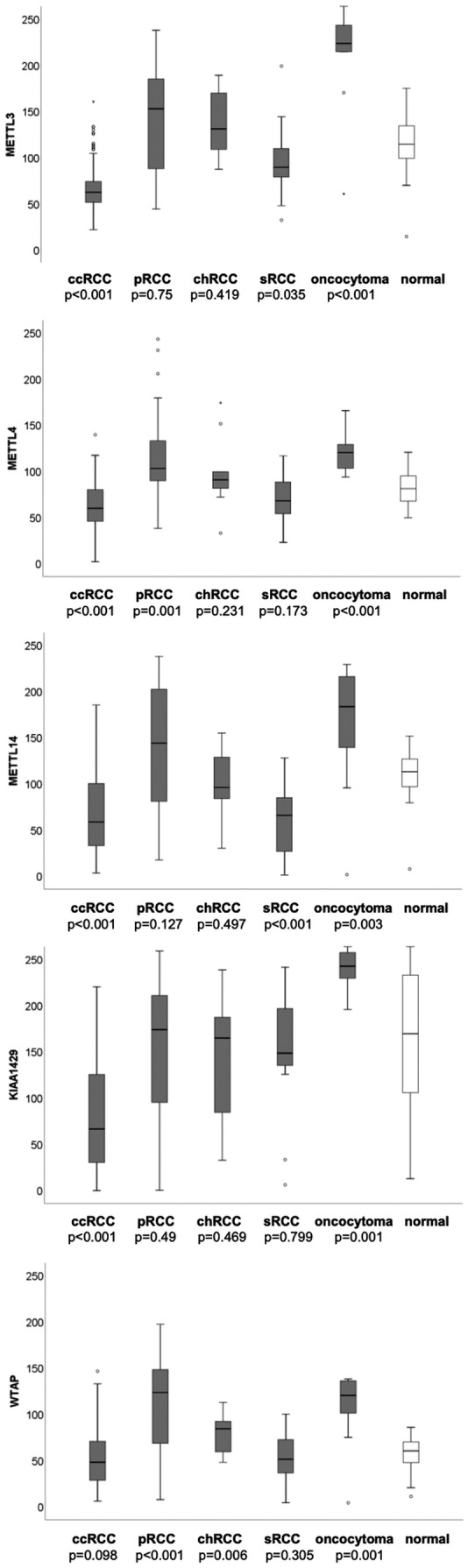
Protein expression of m^6^A‐methyltransferases in renal cell carcinoma subtypes, oncocytoma, and normal renal tissue. Protein expression is dysregulated in RCC subtypes in comparison with normal renal tissue

**FIGURE 6:**
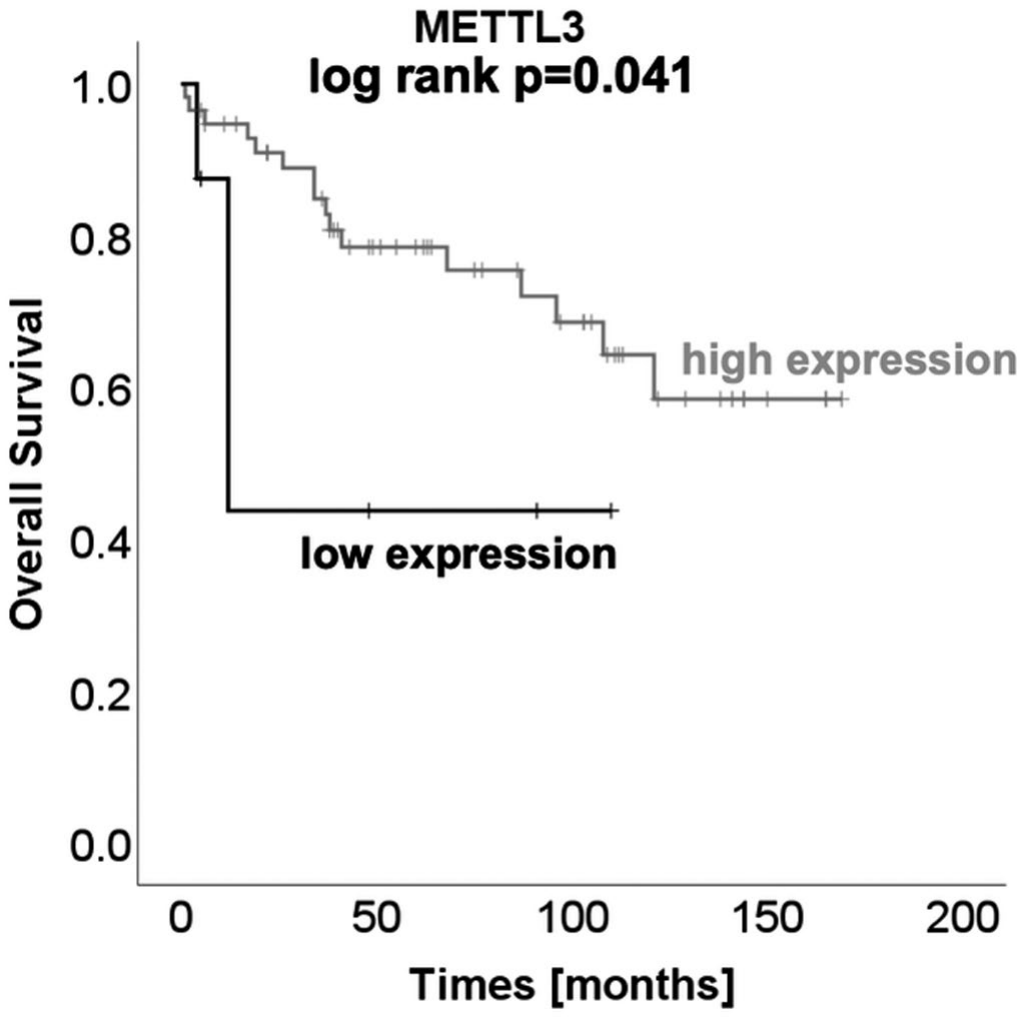
Kaplan–Meier estimates indicate a shortened overall survival in patients with ccRCC and decreased METTL3 protein expression

**FIGURE 7:**
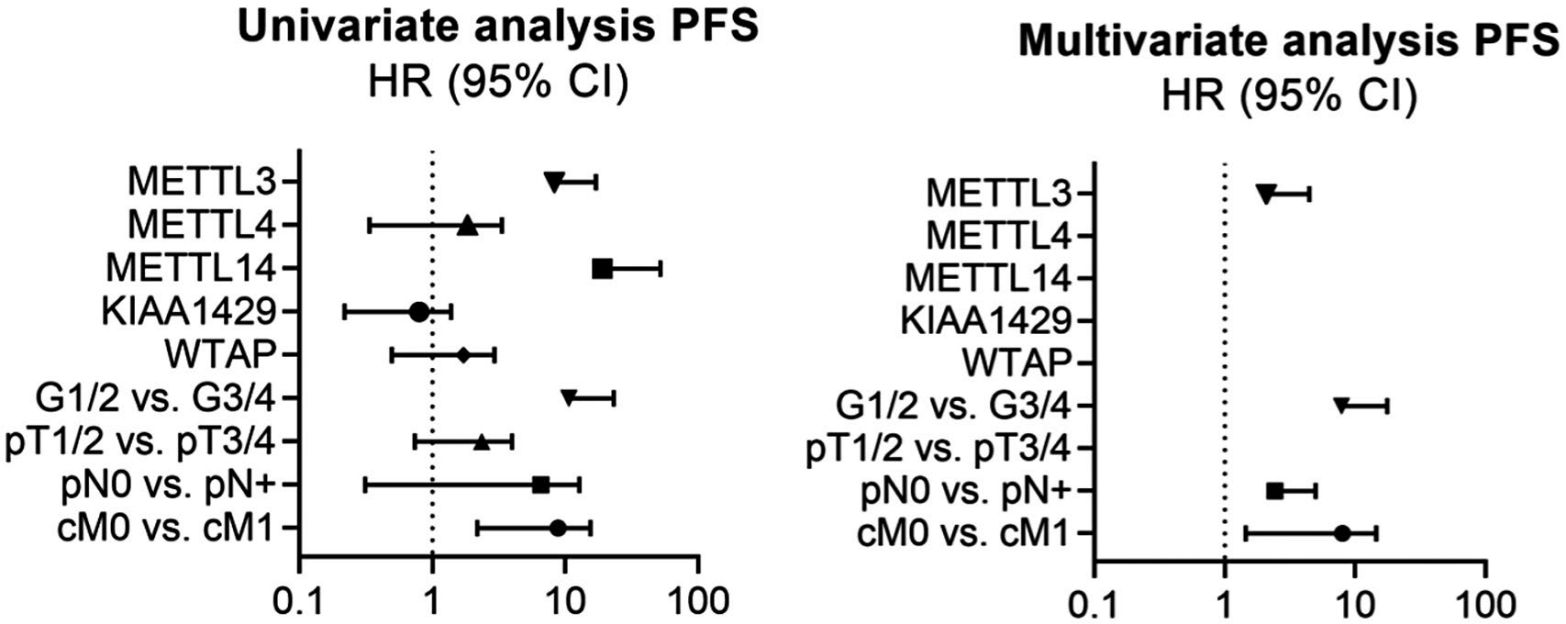
Forest plot analysis of univariate and multivariate Cox regression analyses of the TMA cohort for prediction of progression‐free survival

Immunohistochemical staining was performed to determine the m^6^A‐methyltransferases’ protein expression using a tissue microarray containing ccRCC (n = 160), pRCC (n = 35), chRCC (n = 10), sRCC (n = 16), oncocytomas (n = 10), and normal renal parenchyma samples (n = 30). As expected, protein levels of all methyltransferases except for WTAP were decreased in ccRCC compared to benign tissue, (Mann–Whitney *U*‐test, METTL3, METTL4, METTL14, KIAA1429, all *P* < .001; WTAP *P* = .098). Among the other RCC subtypes methyltransferases’ expressions varied (Mann–Whitney *U*‐test); see Figure 5. Compared to normal renal tissue, protein expression of METTL3 and METTL14 was decreased in sRCC (METTL3 *P* = .035, METTL14 *P* < .001). Protein expression of METTL4 and WTAP was increased in pRCC (METTL4 *P* = .001, WTAP *P* < .001). Protein levels of WTAP were increased in chRCC (*P* = .006). Compared to normal renal tissue protein expression of all investigated methyltransferases was increased in oncocytoma (METTL3 *P* < .001, METTL4 *P* < 0,001, METTL14 *P* = .003, KIAA1429 *P* = .001, WTAP *P* = .001). We did not observe any association with other clinicopathological parameters (Mann–Whitney *U*‐test, Kruskal–Wallis *H*‐test, all *P* > .05). Kaplan–Meier estimates indicated a shorter overall survival in patients with ccRCC and low METTL3 protein expression (log‐rank *P* = .041, see Figure 6), see also detailed information in Supporting Information Table S13. Cox regression analyses also demonstrated that low METTL3 expression levels were predictive for shortened progression‐free survival (METTL3, *P* = .008; *HR (95% CI)* 5.278 (1.541‐18.076)) in the univariate but not multivariate model, see Figure 7 and Supporting Information Table S14. The remaining parameters did not reach statistical significance in the univariate Cox regression analysis (METTL4, METTL14, KIAA1429, and WTAP, all *P* > .05), and therefore they were not included in the multivariate model. See Figure 7 and Supporting Information Table S14. We reviewed our findings with the analysis of the bias‐corrected and accelerated (BCa) bootstrap interval for progression‐free survival; for detailed information see Supporting Information Table S15.

## 4 DISCUSSION

The m^6^A modification influences almost every step of RNA metabolism: generally speaking, m^6^A modification of RNA fastens the RNA’s way from maturation to decomposition. It is involved in mRNA processing, splicing, exporting mRNA from nucleus to cytoplasm, mRNA translation, and decay.^13^, ^14^, ^15^, ^23^, ^24^, ^25^ Interestingly, the m^6^A modification can either play an oncogenic or tumor‐suppressing role in human malignancies depending on the cellular context.^26^, ^27^ The m^6^A methylation is mediated by a multiprotein methylases complex composed of the methyltransferases METTL3, METTL4, METTL14, KIAA1429, and WTAP, either acting as catalyzing subunits or as auxiliary cofactors.^9^, ^11^, ^14^, ^15^, ^28^ The methyltransferases METTL3 and METTL14 build a stable heterodimer core complex, the main part of the multiprotein methyltransferase complex, which is predominantly localized in nuclear speckles, regions enriched with pre‐mRNA in processing to become mature mRNA.^25^, ^29^, ^30^

METTL3, an S‐adenosyl‐L‐methionine (SAM)‐binding subunit of the multiprotein methyltransferase complex, has been detected in the nucleus and cytoplasm,^14^ but was localized predominantly in nuclear speckles.^25^, ^30^ It has a major catalytic methyltransferase activity, by changing its localization from nucleus to cytoplasm, METTL3 can gain a function as a reader, thus also being able to promote translation of target mRNAs.^24^, ^30^ METTL3 may acts as oncogene or as tumor suppressor, depending on the cellular context.^27^ In adenocarcinoma of the lung,^25^ acute myeloid leukemia,^31^ and glioma,^32^ upregulation of METTL3 and increased m^6^A levels have been observed. In contrast, in breast^33^ and colorectal cancer,^34^ the expression of METTL3 inhibited cancer cell viability and proliferation. In our study, a decrease of METTL3 was associated with unfavorable clinical‐pathological parameters and shortened survival following nephrectomy. This finding is supported by others, who observed advanced grade^29^ and shortened overall survival^27^, ^35^ in patients with low METTL3 expression.

A close homolog of METTL3 and component of the multiprotein methyltransferase complex is METTL14.^14^ METTL14 itself shows no catalytic activity, but stabilizes METTL3, maintaining complex integrity, and facilitates RNA binding.^36^, ^37^, ^38^ The stable METTL3‐METTL14 heterodimer core complex has an increased methyltransferase activity compared to each protein individually.^14^, ^39^ Our study demonstrates the downregulation of METTL14 in ccRCC, and low METTL14 expression go along with a shortened survival. Downregulation of METTL14 was also reported in glioma,^40^ whereas high expression levels in acute myeloid leukemia cells enhanced leukemogenesis.^41^

The Wilms tumor‐1 associated protein (WTAP) is involved in the transcriptional and posttranscriptional regulation of cellular genes. It is ubiquitously localized in nuclear speckles and cytoplasm, and partially colocalized with splicing factors.^29^, ^42^, ^43^ WTAP acts as an adaptor molecule in the multiprotein methyltransferase complex, interacting with the METTL3‐METTL14 heterodimer by facilitating its transport into nuclear speckles,^11^, ^14^, ^15^, ^24^ yet itself lacks methyltransferase activity.^29^, ^39^ Recent studies postulated that it might not only be mediating methylation as a writer, but also act as a reader binding mRNA.^44^ Nevertheless, WTAP is required for efficient methylation of mRNA with m^6^A: its knockdown decreased the m^6^A levels in cellular mRNAs even more effectively than knockdown of either METTL3 or METTL14.^39^, ^44^ Even though WTAP only plays an auxiliary part, dysregulation also plays an important role for tumor progression in various malignancies^45^: an oncogenic role was described in lung^46^ and pancreatic cancer.^47^ In contrast, downregulation of WTAP was associated with poor survival in cancer of the bladder, eye, and soft tissue.^46^ Our study demonstrates downregulation of WTAP in ccRCC, and a shortened survival related to low expression levels of WTAP.

The RNA‐binding protein KIAA1429 became of interest for research due to its biochemically shown interaction with WTAP in *Drosophila* concerning sex‐specific splicing.^48^ It is an interacting part of the multiprotein methyltransferase complex.^49^ KIAA1429 is localized in nuclear speckles similar to WTAP.^44^ Knockdown of KIAA1429 causes decreased m6A levels, even more distinctively decreased than depletion observed by the knockdown of METTL3 or METTL4 individually, thereby underlining KIAA1429’s pivotal role in the multiprotein methyltransferase complex.^44^ Several studies propose that KIAA1429 promotes tumorigenesis and progression, for example, in breast cancer^50^ and hepatocellular carcinoma.^49^, ^51^ We demonstrate that KIAA1429 is downregulated in ccRCC, and low expression levels are associated with shortened survival times following nephrectomy.

Although METTL4 is a close homolog of METTL3 and METTL14,^39^, ^52^ and part of the multiprotein methyltransferases complex, it is less studied. Recent studies reported different localizations of METTL4 — either mitochondria or nucleus — thus its functions seem to be context‐dependent: when localized in mitochondria, METTL4 catalyzes N6‐methyldeoxyadenosine (6mA)  ​ methylation of mitochondrial DNA (mtDNA), thereby influencing mitochondrial transcription leading to a reduced mtDNA copy number. Nuclear localization of METTL4 affects the splicing regulation of small nuclear RNAs.^53^ Knockdown of METTL4 did not change cellular m^6^A levels^38^, ^52^; however, it seems to be involved in carcinogenesis. METTL4 is downregulated in colon adenocarcinoma^54^ and upregulated in melanoma.^28^ Further, METTL4 knockdown inhibited melanoma growth.^28^ In ccRCC, we observed a decrease of METTL4, and low METTL4 mRNA expression was indicative of shortened survival.

In conclusion, the m^6^A methyltransferases METTL3, METTL14, WTAP, KIAA1429, and METTL4 are dysregulated in ccRCC and might act as tumor suppressor genes. Our findings demonstrate that patients with low m^6^A methyltransferase expression showed a poor outcome in progression‐free survival, cancer‐specific survival, and overall survival. The optimal cut‐off values utilized for survival analysis were determined using receiver operator characteristic curve analysis, which explains the individual cut‐off values for each methyltransferase in each survival analysis. It should be noted that such kind of cut‐offs needs validation in future studies. Though, the findings of our study are limited by non‐significant results of the multivariate model of Cox regression analyses, partially non‐significant bootstrap analyses, and a finite amount of tissue samples for RCC subtypes. Further investigation including a larger cohort of patients might be necessary to strengthen the conclusion of this study. Keeping these limitations in mind, these findings might not yet enable individualized diagnostic and therapeutic options for patients with ccRCC, still they help to broaden the foundation of a more profound understanding of renal cell carcinomas, and might affect disease management.

Another interesting point worth looking further into is the possible difference in methyltransferases’ expression observed between pRCC and ccRCC. While protein expression of METTL3, METTL4, METTL14, and KIAA1429 was decreased in ccRCC, an increase of expression was observed in METTL4 and WTAP in pRCC, and METTL3 and METTL14, that did not show significance, yet showed a similar trend, see Figure 5. The possible difference might be related to the distinct physiology of these two subtypes. In comparison to ccRCC, which usually presents as a solitary, lipid‐rich tumor with neoplastic cells with clear to sparsely eosinophilic cytoplasm, and a hypervascular blood supply, the papillary subtype manifests multifocally, hypovascular, and in two cytologically and genetically various subtypes with tumor cells varying from barely amphophilic cytoplasm to prominently eosinophilic.^55^ With the small number of pRCC in this study, our results are limited. Thus, it would be of great interest for prospective research to further investigate the difference of regulation and expression of methyltransferases in pRCC in comparison to ccRCC.

## CONFLICT OF INTEREST

The authors declare that there is no conflict of interest.

## Supporting information

Supplementary Material
