# Supplementary material for: Systematic expression analysis of m6A RNA methyltransferases in clear cell renal cell carcinoma
Source: BJUI Compass. 2021 May 3;2(6):402–11. doi: 10.1002/bco2.89 (PMC8988738; doi:10.1002/bco2.89)
Supplement: Supplementary file 1 — Supplementary Material [file BCO2-2-402-s001.docx]

**Supplementary Information**

Authors: L. Gundert, A. Strick, F. von Hagen, D. Schmidt, N. Klümper, Y. Tolkach, M. Toma, G. Kristiansen, M. Ritter, J. Ellinger

Title: Systematic expression analysis of m6A RNA methyltransferases in clear cell renal cell carcinoma

**Supplementary Figure S1:**

Representative photographs of immunohistochemical staining of m6A writers in normal renal and ccRCC tissue.


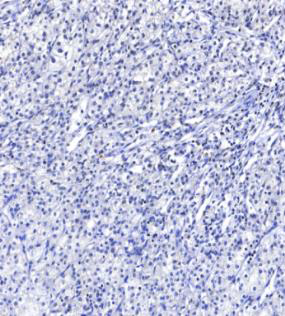
 normal ccRCC normal ccRCC

METTL3


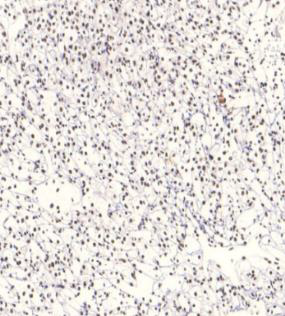

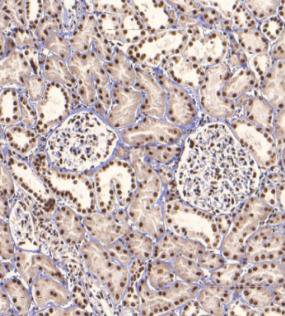


KIAA1429

WTAP

METTL4


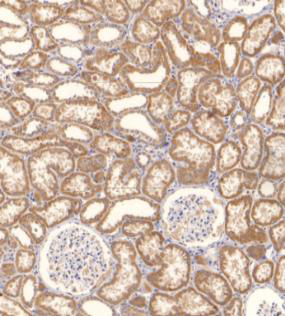

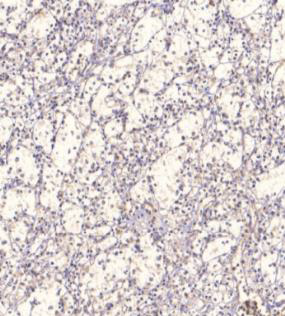


METTL14

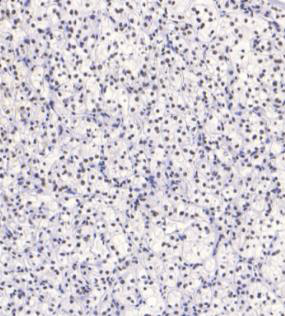
 **Supplementary Table S1:**

Clinical-pathological parameters of the PCR cohort.

| **Characteristics** | **PCR cohort** | |
| --- | --- | --- |
| **Number of patients (%)** | **ccRCC 166 (61.9)** | **Normal 102 (38.1)** |
| Sex, *n* (%)  Male  Female  Age  years, mean (range)  pT Stage, n (%)  pT1  pT2  pT3  pT4  N Stage, *n* (%)  pN0  pN1  Distant metastasis, *n* (%)  M0  M1  Grade, *n* (%)  Grade 1  Grade 2  Grade 3  Grade 4 | 108 (65.1) 58 (34.9)  64.5 (27 - 89)  95 (57.2) 16 (9.6) 52 (31.3) 3 (1.8)  161 (97) 5 (3)  144 (86.7) 22 (13.3)  21 (12.7) 108 (65.1) 28 (16.9) 9 (5.4) | 71 (69.6) 31 (30.4)  63.6 (36 – 89)  -  - - -  - -  - -  - - - - |

**Supplementary Table S2:**

Primer sequences used for real-time PCR experiments.

| **Gene** | **Forward Primer 5’-3’** | **Reverse Primer 3’-5’** |
| --- | --- | --- |
| METTL3  METTL14  WTAP  KIAA1429  METTL4  ACTB  GAPDH  PPIA | CAGGCTCAACATACCCGTACT  TAGCCGCTTGCAGGAGAT  GGAAGTTTACGCCTGATAGCC  TAGCCGCTTGCAGGAGAT  CACCGCTTGCTGAGGTTTTA  CCAACCGCGAGAAGATGA  CTCTGCTCCTCCTGTTCGAC  ATGCTGGACCCAACACAAAT | ACATTCTCTCCCCAACTACA  CACTTTCAGCTCCCAACTGC  CAGCTGCCTTCCAAGCTCT  CATTTCAGCTCCCAACT  TTGCCCCAACTAGTCCAACC  CCAGAGGCGTACAGGGATAG  ACGACCAAATCGGTTGACTC  TCTTTCACTTTGCCAAACACC |

**Supplementary Table S3**

Clinical-pathological parameters of the TMA cohort.

| **Characteristics** | **TMA cohort** | | | | | |
| --- | --- | --- | --- | --- | --- | --- |
| **Number of patients (%)** | **ccRCC 160 (61.3)** | **pRCC 35 (13.4)** | **chRCC 10 (3.8)** | **sRCC 16 (6.1)** | **Oncocytoma 10 (3.8)** | **Normal 30 (11.5)** |
| Sex, *n* (%)  Male  Female  Age, years  Mean   Range  pT Stage, *n* (%)  pT1  pT2  pT3  pT4  n.a.  N Stage, *n* (%)  pN0/pNX  pN+  n.a.  Distant metastasis, *n* (%)  M0/MX  M1  n.a.  Grade, *n* (%)  Grade 1  Grade 2  Grade 3  Grade 4  n.a. | 104 56  61.8 26 - 85  59 (36.9) 31 (19.4) 49 (30.6) 2 (1.3) 19 (11.9)  133 (83.1) 8 (5.1) 19 (11.9)  123 (76.9) 18 (11.3) 19 (11.9)  45 (28.1) 93 (58.1) 3 (1.9) 0 19 (11.9) | 32 3  60.4 35 - 82  20 (57.1) 6 (17.1) 5 (14.3) 0 4 (11.4)  30 (85.7) 1 (2.9) 4 (11.4)  28 (80) 3 (8.6) 4 (11.4)  12 (34.3) 17 (48.6) 2 (5.7) 0 4 (11.4) | 6 4  63.2 27 - 85  6 (60) 4 (40) 0 0 0  10 (100) 0 0  10 (100) 0 0  3 (30) 7 (70) 0 0 0 | 12 4  63.3 51 - 75  0 1 (6.3) 8 (50) 1 (6.3) 6 (37.5)  5 (31.3) 5 (31.3) 6 (37.5)  4 (25) 6 (37.5) 6 (37.5)  0 1 (6.3) 7 (43.8) 2 (12.5) 6 (37.5) | 0 10  57.6 26 - 73  0 0 0 0 10 (100)  0 0 10 (100)  0 0 10 (0)  0 0 0 0 10 (100) | 21 9  57.9 28 - 80  - - - - -  - - -  - - -  - - - - - |
| *n.a.*, not available |  |  |  |  |  |  |

**Supplementary Tables S4:**

Cut-off values for analysis of the PCR cohort for progression-free survival, percent and mean survival of groups higher/lower than the cut-off values.

| **Progression free survival, PCR cohort** | | | | | | |
| --- | --- | --- | --- | --- | --- | --- |
| **Methyltransferases** | **Cut-off** | **Sample amount, *n* (%)** | | | **Mean survial [months]** | |
|  |  | **total** | **< cut-off** | **> cut-off** | **< cut-off** | **> cut-off** |
| **METTL3 **** | **1.2015** | - | - | - | - | - |
| **METTL4 **** | **1.1311** | - | - | - | - | - |
| **METTL14** | **1.1980** | 153 (100) | 55 (36.0) | 98 (64.0) | 150.9 | 79.5 |
| **KIAA1429** | **1.2370** | 153 (100) | 55 (36.0) | 98 (64.0) | 119.3 | 189.4 |
| **WTAP** | **1.2140** | 153 (100) | 81 (52.9) | 72 (47.1) | 157.0 | 81.8 |

The best cut-off was determined based on ROC analyses/ Youden’s Index for m^6^A writers’ expression.

** Kaplan-Meier estimates were not significant (p>0.05).

**Supplementary Tables S5:**

Cut-off values for analysis of the PCR cohort for cancer-specific survival, percent and mean survival of groups higher/lower than the cut-off values.

| **Cancer specific survival, PCR cohort** | | | | | | |
| --- | --- | --- | --- | --- | --- | --- |
| **Methyltransferases** | **Cut-off** | **Sample amount, *n* (%)** | | | **Mean survival [months]** | |
|  |  | **total** | **< cut-off** | **> cut-off** | **< cut-off** | **> cut-off** |
| **METTL3** | **1.2556** | 154 (100) | 54 (35.1) | 100 (64.9) | 173.6 | 180.1 |
| **METTL4** | **1.2632** | 154 (100) | 53 (34.4) | 101 (65.6) | 172.7 | 173.2 |
| **METTL14** | **1.2882** | 154 (100) | 57 (37.0) | 97 (63.0) | 166.9 | 87.1 |
| **KIAA1429** | **1.4190** | 154 (100) | 25 (16.2) | 129 (83.7) | 103.9 | 195.3 |
| **WTAP** | **1.3560** | 154 (100) | 48 (31.1) | 106 (68.8) | 124.3 | 208.7 |

The best cut-off was determined based on ROC analyses/ Youden’s Index for m^6^A writers’ expression.

**Supplementary Tables S6:**

Cut-off values for analysis of the PCR cohort for overall survival, percent and mean survival of groups higher/lower than the cut-off values.

| **Overall survival, PCR cohort** | | | | | | |
| --- | --- | --- | --- | --- | --- | --- |
| **Methyltransferases** | **Cut-off** | **Sample amount, *n* (%)** | | | **Mean survival [months]** | |
|  |  | **total** | **< cut-off** | **> cut-off** | **< cut-off** | **> cut-off** |
| **METTL3** | **1.2654** | 154 (100) | 22 (14.3) | 132 (85.7) | 78.9 | 174.2 |
| **METTL4** | **1.2806** | 154 (100) | 40 (26.0) | 114 (74.0) | 104.8 | 170.9 |
| **METTL14** | **1.2811** | 154 (100) | 30 (19.5) | 124 (80.5) | 93.4 | 186.6 |
| **KIAA1429** | **1.3603** | 154 (100) | 60 (39.0) | 94 (61.0) | 108.6 | 178.3 |
| **WTAP** | **1.2560** | 154 (100) | 48 (31.2) | 106 (68.8) | 111.0 | 167.7 |

The best cut-off was determined based on ROC analyses/ Youden’s Index for m^6^A writers’ expression.

**Supplementary Table S7:**

Univariate and multivariate Cox regression analysis of the PCR cohort for the prediction of progression-free survival in ccRCC patients.

| **Progression-free survival,**  **PCR cohort** | **Univariate analysis** | | **Multivariate analysis** | |
| --- | --- | --- | --- | --- |
|  | ***p* value** | **HR (95% CI)** | ***p* value** | **HR (95% CI)** |
| Expression of m6A methyltransferases*  High (> cut-off)  Low (< cut-off)  METTL3  METTL4  METTL14  KIAA1429  WTAP  Clinicopathological parameters  Grading grouped  G1/2  G3/4  pT Stage grouped  pT1/2  pT3/4  pN Stage grouped  pN0  pN+  pM Stage   M0  M1 | 0.075 0.107 0.046 0.010 0.047  0.001  <0.001  0.069  <0.001 | 1.00  1.860 (0.939 - 3.683) 1.877 (0.872 - 4.040) 2.004 (1.011 - 3.971) 2.458 (1.238 - 4.880) 2.121 (1.009 - 4.458)  1.00 3.088 (1.544 - 6.175)  1.00 5.152 (2.518 - 10.540)  1.00 3.780 (0.901 - 15.854)  1.00 6.463 (3.039 - 13.746) | - - 0.836 0.223 0.356  0.255  0.002  -  0.043 | 1.00  - - 1.099 (0.449 - 2.692) 1.720 (0.719 - 4.114) 1.499 (0.635 - 3.541)  1.00 1.619 (0.707 - 3.707)  1.00 3.544 (1.592 - 7.888)  - -  1.00 2.737 (1.033 - 7.254) |

*HR* – Hazard ratio; *95% CI* – 95%-Confidence Interval, *Dichotomization is based on ROC analyses/ Youden’s Index as best cut-off for m^6^A writers’ expression

**Supplementary Table S8:**

Univariate and multivariate Cox regression analysis of the PCR cohort for the prediction of cancer-specific survival in ccRCC patients.

| **Cancer-specific survival,**  **PCR cohort** | **Univariate analysis** | | **Multivariate analysis** | |
| --- | --- | --- | --- | --- |
|  | ***p* value** | **HR (95% CI)** | ***p* value** | **HR (95% CI)** |
| Expression of m6A methyltransferases*  High (> cut-off)  Low (< cut-off)  METTL3  METTL4  METTL14  KIAA1429  WTAP  Clinicopathological parameters  Grading grouped  G1/2  G3/4  pT Stage grouped  pT1/2  pT3/4  pN Stage grouped  pN0  pN+  pM Stage   M0  M1 | 0.051 0.037 0.048 <0.001 0.004  0.001  0.006  0.035  <0.001 | 1.00  2.365 (0.995 - 5.621) 2.515 (1.056 - 5.989) 2.463 (1.007 - 6.023) 6.644 (2.814 - 15.687) 3.606 (1.490 - 8.729)  1.00 3.216 (1.329 - 7.780)  1.00 3.356 (1.410 - 7.985)  1.00 4.848 (1.116 - 21.070)  1.00 8.602 (3.644 - 20.304) | 0.331 0.926 0.871 0.008 0.354  0.267  0.448  0.594  0.001 | 1.00  1.574 (0.631 - 3.928) 0.941 (0.261 - 3.393) 0.893 (0.226 - 3.531) 4.635 (1.479 - 14.523) 1.771 (0.529 - 5.926)  1.00 1.744 (0.654 - 4.651)  1.00 1.499 (0.527 - 4.262)  1.00 0.647 (0.130 - 3.209)  1.00 6.198 (2.118 - 18.134) |

*HR* – Hazard ratio; *95% CI* – 95%-Confidence Interval, *Dichotomization is based on ROC analyses/ Youden’s Index as best cut-off for m^6^A writers’ expression

**Supplementary Tables S9:**

Univariate and multivariate Cox regression analysis of the PCR cohort for the prediction of overall survival in ccRCC patients.

| **Overall survival,**  **PCR cohort** | **Univariate analysis** | | **Multivariate analysis** | |
| --- | --- | --- | --- | --- |
|  | ***p* value** | **HR (95% CI)** | ***p* value** | **HR (95% CI)** |
| Expression of m6A methyltransferases*  High (> cut-off)  Low (< cut-off)  METTL3  METTL4  METTL14  KIAA1429  WTAP  Clinicopathological parameters  Grading grouped  G1/2  G3/4  pT Stage grouped  pT1/2  pT3/4  pN Stage grouped  pN0  pN+  pM Stage   M0  M1 | 0.002 0.016 0.022 0.004 0.031  0.039  <0.001  0.008  <0.001 | 1.00  3.272 (1.563 - 6.851) 2.395 (1.177 - 4.874) 2.334 (1.129 - 4.825) 3.059 (1.435 - 6.517) 2.19 (1.074 - 4.466)  1.00 2.279 (1.044 - 4.975)  1.00 3.629 (1.771 - 7.437)  1.00 5.150 (1.541 - 17.213)  1.00 5.763 (2.709 - 12.261) | 0.083 0.469 0.427 0.108 0.422  0.592  0.045  0.971  0.01 | 1.00  2.194 (0.901 - 5.340) 1.480 (0.512 - 4.278) 0.598 (0.168 - 2.129) 2.180 (0.843 - 5.642) 1.427 (0.600 - 3.396)  1.00 1.272 (0.528 - 3.063)  1.00 2.315 (1.020 - 5.251)  1.00 0.975 (0.245 - 3.878)  1.00 3.429 (1.344 - 8.749) |

*HR* – Hazard ratio; *95% CI* – 95%-Confidence Interval, *Dichotomization is based on ROC analyses/ Youden’s Index as best cut-off for m^6^A writers’ expression

**Supplementary Tables S10:**

Bootstrap analysis of univariate and multivariate Cox regression analysis of the PCR cohort for the prediction of progression-free survival in ccRCC patients.

| **Bootstrap analysis** | | | | | | |
| --- | --- | --- | --- | --- | --- | --- |
| **Progression-free survival,**  **PCR cohort** | **Univariate analysis** | | | **Multivariate analysis** | | |
|  | ***p* value** | **Bootstrap BCa (95% CI)** | | ***p* value** | **Bootstrap Bca (95% CI)** | |
|  |  | **lower** | **upper** |  | **lower** | **upper** |
| METTL3  METTL4  METTL14  KIAA1429  WTAP  G1/2 vs. G3/4  pT1/2 vs. pT3/4  N0 vs. N+  cM0 vs. cM1 | 0.002  0.017  0.020  0.004  0.029  0.038  0.002  0.028  0.001 | 0.424  0.065  0.149  0.326  -0.012  -0.041  0.585  -3.03  0.882 | 1.884  1.639  1.473  2.071  1.516  1.614  2.032  3.499  2.549 | 0.045  0.487  0.489  0.111  0.424  0.648  0.061  0.910  0.019 | -0.150  -0.920  -1.937  -0.597  -0.617  -1.224  -0.191  -13.309  -0.114 | 1.812  1.519  1.094  2.465  1.251  1.445  1.820  2.208  2.583 |

Bootstrap analysis was performed using the bias-corrected and accelerated (BCa) bootstrap interval.

**Supplementary Tables S11:**

Bootstrap analysis of univariate and multivariate Cox regression analysis of the PCR cohort for the prediction of cancer-specific survival in ccRCC patients.

| **Bootstrap analysis** | | | | | | |
| --- | --- | --- | --- | --- | --- | --- |
| **Cancer-specific survival,**  **PCR cohort** | **Univariate analysis** | | | **Multivariate analysis** | | |
|  | ***p* value** | **Bootstrap Bca (95% CI)** | | ***p* value** | **Bootstrap Bca (95% CI)** | |
|  |  | **lower** | **upper** |  | **lower** | **upper** |
| METTL3  METTL4  METTL14  KIAA1429  WTAP  G1/2 vs. G3/4  pT1/2 vs. pT3/4  N0 vs. N+  cM0 vs. cM1 | 0.036  0.033  0.031  0.001  0.003  0.004  0.005  0.051  0.001 | 0.014  0.007  -0.071  0.915  0.304  0.222  0.167  -3.047  1.313 | 1.880  1.960  2.005  3.009  2.494  2.081  2.380  3.514  2.963 | 0.359  0.922  0.845  0.019  0.246  0.347  0.489  0.54  0.008 | -0.638  -1.493  -1.661  -0.294  -0.710  -1.281  -1.167  -14.662  0.18 | 1.556  1.182  1.794  5.975  1.664  2.043  1.896  2.374  3.573 |

Bootstrap analysis was performed using the bias-corrected and accelerated (BCa) bootstrap interval.

**Supplementary Tables S12:**

Bootstrap analysis of univariate and multivariate Cox regression analysis of the PCR cohort for the prediction of overall survival in ccRCC patients.

| **Bootstrap analysis** | | | | | | |
| --- | --- | --- | --- | --- | --- | --- |
| **Overall survival,**  **PCR cohort** | **Univariate analysis** | | | **Multivariate analysis** | | |
|  | ***p* value** | **Bootstrap Bca (95% CI)** | | ***p* value** | **Bootstrap Bca (95% CI)** | |
|  |  | **lower** | **upper** |  | **lower** | **upper** |
| METTL3  METTL4  METTL14  KIAA1429  WTAP  G1/2 vs. G3/4  pT1/2 vs. pT3/4  N0 vs. N+  cM0 vs. cM1 | 0.075  0.114  0.047  0.003  0.045  0.001  0.001  0.001  0.001 | -0.109  -0.245  0.014  0.224  -0.009  0.367  0.960  -3.031  1.110 | 1.380  1.327  1.456  1.619  1.745  1.887  2.483  1.891  2.588 | 0.440  0.768  0.826  0.228  0.331  0.354  0.003  0.247  0.15 | -0.493  -1.018  -0.792  -0.401  -0.604  -0.951  0.18  -12.111  -0.568 | 1.023  1.184  1.149  1.480  1.425  1.748  2.513  0.443  2.518 |

Bootstrap analysis was performed using the bias-corrected and accelerated (BCa) bootstrap interval.

**Supplementary Tables S13:**

Cut-off values for analysis of the TMA cohort for overall survival, percent and mean survival of groups higher/lower than the cut-off values.

| **Overall survival, TMA cohort** | | | | | | |
| --- | --- | --- | --- | --- | --- | --- |
| **Methyltransferases** | **Cut-off** | **Sample amount, *n* (%)** | | | **Mean survival [months]** | |
|  |  | **total** | **< cut-off** | **> cut-off** | **< cut-off** | **> cut-off** |
| **METTL3** | **1.3693** | 65 (100) | 57 (87.7) | 8 (12.3) | 124.1 | 53.9 |
| **METTL4 **** | **1.3079** | - | - | - | - | - |
| **METTL14 **** | **1.2667** | - | - | - | - | - |
| **KIAA1429 **** | **1.2886** | - | - | - | - | - |
| **WTAP **** | **1.1851** | - | - | - | - | - |

The best cut-off was determined based on ROC analyses/ Youden’s Index for m^6^A writers’ expression.

** Kaplan-Meier estimates were not significant (p>0.05).

**Supplementary Table S14:**

Univariate and multivariate Cox regression analysis of the TMA cohort for the prediction of progression-free survival in ccRCC patients.

| **Progression-free survival, TMA cohort** | **Univariate analysis** | | **Multivariate analysis** | |
| --- | --- | --- | --- | --- |
|  | ***p* value** | **HR (95% CI)** | ***p* value** | **HR (95% CI)** |
| Expression of m6A methyltransferases*  High (> cut-off)  Low (< cut-off)  METTL3  METTL4  METTL14  KIAA1429  WTAP  Clinicopathological parameters  Grading grouped  G1/2  G3/4  pT Stage grouped  pT1/2  pT3/4  pN Stage grouped  pN0  pN+  pM Stage   M0  M1 | 0.008 0.436 0.395 0.293 0.359  0.022  0.066  0.006  <0.001 | 1.00  5.278 (1.541 - 18.076) 1.428 (0.583 - 3.498)  0.45 (<0.001 - 57.115) 0.658 (0.301 - 1.436) 1.427 (0.667 - 3.052)  1.00 5.677 (1.289 - 25.007)  1.00 1.988 (0.957 - 4.132)  1.00 4.561 (1.540 - 13.511)  1.00 7.160 (3.171 - 16.165) | 0.819 - - - -  0.106  -  0.513  <0.001 | 1.00  1.177 (0.29 - 4.773) - - - -  1.00 3.785 (0.755 - 18.986)  - -  1.00 1.519 (0.435 - 5.309)  1.00 6.214 (2.528 - 15.277) |

*HR* – Hazard ratio; *95% CI* – 95%-Confidence Interval, *Dichotomization is based on ROC analyses/ Youden’s Index as best cut-off for m^6^A writers’ expression

**Supplementary Tables S15**

Bootstrap analysis of univariate and multivariate Cox regression analysis of the TMA cohort for the prediction of progression-free survival in ccRCC patients.

| **Bootstrap analysis** | | | | | | |
| --- | --- | --- | --- | --- | --- | --- |
| **Progression-free survival,**  **TMA cohort** | **Univariate analysis** | | | **Multivariate analysis** | | |
|  | ***p* value** | **Bootstrap BCa (95% CI)** | | ***p* value** | **Bootstrap BCa (95% CI)** | |
|  |  | **lower** | **upper** |  | **lower** | **upper** |
| METTL3  METTL4  METTL14  KIAA1429  WTAP  G1/2 vs. G3/4  pT1/2 vs. pT3/4  N0 vs. N+  cM0 vs. cM1 | 0.002  0.406  0.001  0.307  0.358  0.038  0.041  0.006  0.001 | 0.801  -0.586  -3.24  -1.148  -0.464  -4.407  -1.379  -2.857  -2.868 | 3.384  1.153  -3.031  0.332  1.140  3.048  0.045  -0.049  -1.298 | 0.098  0.869  0.017  0.188  0.128  0.015  0.281  0.645  0.002 | -0.323  -1.277  -15.035  -3.009  -0.605  -3.331  -1.236  -1.673  -3.007 | 4.676  1.401  -11.178  0.431  3.133  11.384  0.229  1.943  0 |

Bootstrap analysis was performed using the bias-corrected and accelerated (BCa) bootstrap interval.
